# The UPBEAT depression and coronary heart disease programme: using the UK medical research council framework to design a nurse-led complex intervention for use in primary care

**DOI:** 10.1186/1471-2296-13-119

**Published:** 2012-12-12

**Authors:** Elizabeth A Barley, Mark Haddad, Rosemary Simmonds, Zoe Fortune, Paul Walters, Joanna Murray, Diana Rose, André Tylee

**Affiliations:** 1 Florence Nightingale School of Nursing and Midwifery, King’s College London, James Clerk Maxwell Building, 57 Waterloo Road, London, SE1 8WA, UK; 2 School of Health Sciences, City University London, Northampton Square, London, EC1V 0HB, UK; 3 Research Associate, the Centre for Academic Primary Care, School of Social and Community Medicine, University of Bristol, 39 Whatley Road, Clifton, Bristol, BS8 2PS, UK; 4 Research Worker, Section of Primary Care Mental Health, Health Services and Population Research Department, Institute of Psychiatry, King’s College London, PO Box 28, De Crespigny Park, London, SE5 8AF, UK; 5 Consultant Psychiatrist, Weymouth and Portland CMHT, Dorset Healthcare University Foundation NHS Trust, Radipole Lane, Weymouth, Dorset, DT4 0QE, UK; 6 Section of Mental Health and Ageing, Health Services and Population Research Department, Institute of Psychiatry, King’s College London, PO Box 28, De Crespigny Park, London, SE5 8AF, UK; 7 Reader in User-Led Research, Head of Section and Co-director Service User Research Enterprise (SURE), PO34 Institute of Psychiatry, King's College London, De Crespigny Park, London, SE5 8AF, UK

**Keywords:** Complex intervention, Personalised care, Coronary heart disease, Depression, Primary care

## Abstract

**Background:**

Depression is common in coronary heart disease (CHD) and increases the incidence of coronary symptoms and death in CHD patients. Interventions feasible for use in primary care are needed to improve both mood and cardiac outcomes. The UPBEAT-UK programme of research has been funded by the NHS National Institute for Health Research (NIHR) to explore the relationship between CHD and depression and to develop a new intervention for use in primary care.

**Methods:**

Using the Medical Research Council (MRC) guidelines for developing and evaluating complex interventions, we conducted a systematic review and qualitative research to develop a primary care-based nurse-led intervention to improve mood and cardiac outcomes in patients with CHD and depression. Iterative literature review was used to synthesise our empirical work and to identify evidence and theory to inform the intervention.

**Results:**

We developed a primary care-based nurse-led personalised care intervention which utilises elements of case management to promote self management. Following biopsychosocial assessment, a personalised care plan is devised. Nurses trained in behaviour change techniques facilitate patients to address the problems important to them. Identification and utilisation of existing resources is promoted. Nurse time is conserved through telephone follow up.

**Conclusions:**

Application of the MRC framework for complex interventions has allowed us to develop an evidence based intervention informed by patient and clinician preferences and established theory. The feasibility and acceptability of this intervention is now being tested further in an exploratory trial.

## Background

The prevalence of depression in coronary heart disease (CHD) patients has been estimated at 20% [1]. Depression increases the incidence of coronary symptoms and death in CHD patients [1]. Interventions for patients with depression and CHD improve depression outcomes, but there is limited evidence for benefit on cardiac outcomes, with only a single study indicating improvements in disease control [2]. The UPBEAT-UK programme of research [3] has been funded by the National Institute for Health Research (NIHR) to explore the relationship between CHD and depression and to develop a new intervention for use in primary care.

Complex health care interventions are ‘made up from various interconnecting parts’ [4] which act both independently and interdependently making them difficult to design and evaluate. To assist with this, the Medical Research Council (MRC) published a framework first in 2000 [5], then revised in 2008 [6,7] to inform intervention development, feasibility/piloting, evaluation and implementation. These may be thought of as stages, though ‘often these will not follow a linear or even a cyclical sequence’ [6] as one stage may be repeated in order to incorporate findings from other stages. Reporting of the development stage is considered important as this informs implementation and evaluation. The development stage of the MRC guidance involves ‘identifying the evidence base’, ‘identifying or developing theory’ and ‘modelling process and outcomes’. The final modelling phase involves using research to progressively refine the design prior to intervention evaluation [7].

We conducted a series of studies to inform the UPBEAT-UK intervention; these include a systematic review of studies of depression management in the UK [8] and qualitative studies of practice nurses (PNs) and general practitioners (GPs) [9] and of patients with CHD and depression. This paper reports how we combined this series of studies using iterative evidence review and additional qualitative research to develop and model a nurse-led intervention to improve mood and cardiac outcomes in primary care patients with CHD and depression.

## Methods

**Figure 1:**
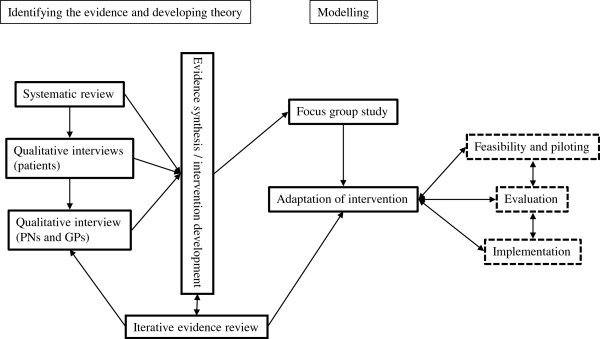
**UPBEAT intervention MRC development stage.** Feasibility and piloting, Evaluation and Implementation stages are ongoing.

This work consists of three phases: 1) studies to inform intervention, including a systematic review and a qualitative study of clinicians and of patients, 2) Integration of findings from the informative studies with iterative evidence review to inform the intervention, 3) Modelling of the intervention, which involved a focus group study and further evidence review. This process is detailed in Figure 1.

### Phase 1: Informative studies

#### Systematic review of studies of depression management in the UK

Full methods have been reported [8]. We searched for qualitative or quantitative studies conducted in the UK and published after 2000 which contained GP or PN generated data concerning attitudes towards and experience of managing depression. Multiple databases were searched and study selection and quality assessment were conducted independently by two authors. Data were synthesised using principles from meta-ethnography [10].

#### Interviews with PNs and GPs to determine preferences for the future intervention

We conducted in-depth interviews with 12 PNs and 10 GPs working in practices in 4 ethnically and culturally diverse South East London boroughs. A purposive, maximum variation approach to recruitment was used based on ethnicity, age, practice setting and type (single handed *versus* group). Interviews were conducted and transcribed verbatim by EB. Analyses were performed concurrently using principles of constant comparison [11] and thematic analysis [12]. Full methodological details have been reported [9]. Original data concerning preferences for the future intervention are reported here.

#### Interviews with patients with CHD and depression

The aim of this study was to determine patient needs and preferences for the future intervention. The sampling frame was the UPBEAT-UK cohort study [3]: a 4-year study of 803 patients with CHD recruited from the Quality and Outcomes Framework CHD registers of 16 GP practices in South London. Thirty patients (19 male) with a positive Patient Health Questionniare-2 (PHQ-2)[13] score were sampled consecutively. They were aged 47 to 85 (mean 65); 21 were white British. Interviews were transcribed verbatim and analysed using a thematic approach. Full details are available from the authors; a paper describing this study is currently under review.

### Phase 2: Integration of findings and iterative evidence review to inform the intervention

Findings from our informative studies were discussed at multidisciplinary project group and independent steering group meetings. Disciplines represented included medicine (psychiatry, cardiology, general practice), nursing (general and mental health) and psychology (clinical and health). Findings were used to guide literature searches which were performed iteratively at each stage of the research. We focused on identifying high quality systematic reviews and evidence based guidelines. We searched the Cochrane Database of Systematic Reviews, the Database of Abstracts of Reviews of Effectiveness and the NICE website. We searched for evidence published subsequently to the reviews and guidelines using Medline, Embase and Psychinfo. Key words included coronary heart disease, angina, myocardial infarction, depression, common mental disorder, behaviour change and primary care. Findings from the evidence reviews were discussed and used to support choices for the intervention content.

### Phase 3: Modelling of the intervention

Focus Group study: The aim of this study was to determine the potential acceptability of the intervention to patients with CHD and depression and to identify whether any changes were necessary. The focus group design was chosen as we felt between participant discussion and group interaction would clarify issues relevant to the broader population, rather than highlighting needs specific to individuals. UPBEAT-UK cohort study [3], participants reporting chest pain (modified Rose Angina Questionnaire [14] and identified as depressed (PHQ2 positive [13]) were identified. Maximum variation sampling was used to select patients varying by gender, age, ethnicity and borough of residence. Two focus groups were held at the Institute of Psychiatry. A short presentation explained the proposed intervention and the intervention materials (patient information leaflet, assessment form, personalised health plan) were provided. All participants gave informed consent and ethical approval was granted, along with that for the other qualitative studies, by Bexley and Greenwich Research Ethics committee (07/H0809/38).

Two researchers (RS and ZF) facilitated the groups and notes of key discussion points (verified by participants) were used to guide the analysis. Discussions were tape recorded and transcribed by RS. Transcripts were entered into NVivo 8 qualitative software [15]. Coding was informed by the aims of the study. The coding frame and themes were discussed within the team.

## Results

### Systematic review of studies of depression management in the UK

Detailed findings, including a PRISMA flow diagram, have been reported [8]. In summary, our review, which included 7 qualitative and 10 quantitative studies, indicated that PNs and GPs are unsure of the nature of the relationship between low mood and social problems and of their role in managing it. Ambivalent attitudes to working with depressed people, a lack of confidence, the use of a limited number of management options and a belief that a diagnosis of depression is stigmatising complicated the management. However, we found no studies of the management of depression co-morbid with physical health problems.

### Interviews with PNs and GPs to determine preferences for the future intervention

Our findings concerning attitudes towards the management of depression in CHD have been reported elsewhere [9]. Essentially, the PNs and GPs viewed depression in CHD as similar to depression without physical co-morbidity. The PNs in particular expressed a lack of confidence in dealing with depression. Depression in CHD was perceived as often associated with psychosocial problems and staff reported uncertainty concerning their management. Concerning preferences for the new intervention, there were two main themes: ‘intervention content preferences’ and ‘who would deliver the intervention’. Quotes are identified by participant profession and interview number (i.e. PN1 to 12, GP1 to 10).

#### Theme: intervention content preferences

##### Personalised care

GPs and PNs recognised that needs vary between individuals and wanted a tailored management strategy incorporating patient preferences.

“That there isn’t a ‘one size fits all’ package, but there’s a range of options, and that decision on tailoring the package is made by….the patient makes the decision with the support of their GP.” (GP3)

“I think there’s got to be lots of different options because everybody’s different.” (PN3)

A personalised approach was viewed as likely to increase adherence to treatment.

“You’re more likely to get sort of concordance with people following if they’ve been given a choice of their options and you’ve explained it to them.” (PN6)

One PN was cynical, appearing to believe that patients could never be satisfied:

“I think it doesn’t matter what you do for some patients, they want more” (PN2)

##### Accessibility

This issue was raised by almost all GP and PN participants. The intervention should be available locally, preferably practice-based, as it was thought that patients disliked having to travel and would be more comfortable somewhere familiar.

“They do complain when they have to go out of the borough” (PN2)

It should also be available outside working hours to accommodate employed patients. Participants also wanted to offer intervention immediately as, when patients with depression have to wait for treatment, clinicians feel powerless.

“It’s not much use having it if there’s gonna be like a 3 month wait. In that interim, what are you doing for the patient?” (GP7)

Simple or no referral criteria were requested as difficulty was reported recalling referral criteria for some interventions, such as counselling services.

“Where you go depends on your age, your postcode, your diagnosis, any number of conditions that make it horribly complex to navigate which must make very little sense if you’re a patient and is pretty bloody annoying if you’re on the professional side.” (GP2)

##### Protocols

Stepped care protocols were more favoured by PNs than GPs. It was suggested by both groups that nurses are trained to follow procedures, whereas GPs prefer to act more independently.

“It would be nice to have a good pathway to follow, rather than ‘oh well, we might do this to this one, that to that one’, so a definite pathway.” (PN2)

“If you had sort of guidelines that you’d develop, it would be good for us to have them, so if we think ‘yeah gosh, look here’s someone and I don’t know what to do’ you can actually look at it. It, it just helps you, it really does help you.” (PN6)

##### Timing

Several participants felt strongly that any intervention should occur soon after a cardiac event believing that early intervention would be more effective.

“What we normally do is invite them in after they’ve finished their cardiac rehab. I try not to invite them while they are still doing it because we’re overlapping in a way…. I can then reinforce what hopefully they’ve learnt through that.” (PN9)

“It is important to, as soon as they are discharged from hospital, to bring them in here and discuss their experiences and explore their understanding of what is going on. And to explore their immediate home and domestic life and their social relationships and see whether they are on that path of distress yet, and if so what can we do to stop them from tipping further because, the further, they tip the more depression is waiting for them.” (GP5)

Others suggested seeing patients some months later to reinforce the messages provided during cardiac rehabilitation. Timing was not discussed in terms of patients with CHD who had not had an event, perhaps because, in these patients, participants feel less able to predict when depression might develop. However, one PN did suggest that a ‘pre heart attack group’ would be useful for patients at high risk.

##### Specific content

A very wide range of interventions was suggested. Several participants were focused on changing lifestyle-related risk factors for CHD. Exercise, smoking and alcohol consumption were mentioned.

“Things like exercise and nice places to go, be, remove stress. And clearly we need to be dealing with other things like smoking, like drinking where that’s appropriate and, you know, - alcohol and depression – not a good combination….” (GP2)

Group therapy based on the elements of cardiac rehabilitation was popular. Peer support groups were cited as being useful in providing empathy and reducing isolation.

“I can do so much, but it’s better if it’s covered in a big group.” (PN7)

A more significant role for psychological services, not just CBT but counselling and chronic disease-specific interventions delivered by psychologists, was also considered important.

“And the psychologist service is below capacity. I’d have that increased. I’d like them to be trained in helping people with chronic diseases.” (GP4)

One GP and one PN talked of the need for interventions that increased feelings of self-efficacy and motivated patients to play an active role in recovery.

“I think it would be better if, as I say I have no idea how you would achieve it, if you could find a way of making people see that actually they can keep themselves well.”(PN5)

One GP and one PN suggested there may be a role for complimentary therapies as many patients favour these.

##### Theme

*Who would deliver a new CHD depression intervention?* Opinions were mixed. Ten participants (4 GPs and 6 PNs) suggested that PNs would be most suitable. PNs compared with GPs were seen as having a better quality relationship with their patients and more experience of managing long term conditions.

“If you sort of see them year in year out, you do form a rapport erm and so you feel comfortable about talking about those issues. ‘Cause it might be a bit inappropriate if you’ve only met someone once and they suddenly come out ‘well are you depressed?” (PN8)

“The GPs aren’t seeing the long term conditions. They only see them for acute things. Yeah, we’re the ones that need to manage that, not them.” (PN6)

GPs and PNs also thought PNs would find satisfaction delivering the intervention:

“They’re an incredibly caring profession. A lot of those caring attributes and values are not tapped into sufficiently by the actual role they are drawn into in general practice.” (GP1)

“A lot of nurses that run chronic diseases clinics will probably feel that their job was more satisfactory by knowing that you provide an additional service, rather than just sort of ticking the box for their payments for QOF.” (PN8)

One GP thought that nurses would be unsuitable, believing that they are not trained to be flexible in their approach to management.

“The nursing training is very rigid, and protocol driven and this is a complete turn off to a large group of this sort of patient.” (GP8)

Others felt that PNs may be suitable, but would need specific training, mental health expertise, supervision or a protocol to help them overcome a perceived lack of knowledge concerning mental health and a focus on physical health-related tasks.

“It would be nice to have a nurse-led one, but I think it would be somebody who would have a specific interest in erm mental health or psychology….I’m much more of a generalist.” (PN2)

“At a CHD clinic assessment there are lists of about probably 15 things that we have to tick off for that patient physically, so to actually try and do anything on the depression front would be very difficult, and I don’t think most of us are trained or equipped to deal with things like that.” (PN1)

Three GPs, based on past experience, felt that a psychologist or counsellor with knowledge of CHD would be best.

“You could maybe have .… a psychologist that specialised in seeing people with CHD, someone who could appreciate the most pressing concerns, would know what the most common mood disturbances are.” (GP7)

“They were psychologists who’d done their degree. They were only **youngsters**, but they were looking at self help leaflets, motivation leaflets .… and she was then developing herself as an exercise counsellor and you could almost see this sort of, this sort of mixture of a motivator, an exercise counsellor, a little bit of understanding about cognitive behavioural things and making some changes and that sort of stuff. So, that would be the sort of ideal person.” (GP8)

Other professionals considered as possibilities were pharmacists, mental health nurses (RMNs), social workers and healthcare assistants.

### Interviews with patients with CHD and depression

This study is currently under review, but we can report that, similar to our other studies, the impact of social problems on patients with both CHD and depression was highlighted (details available from the authors).

### Phase 2: Integration of findings and iterative evidence review to inform the intervention

Data from our informative studies were remarkably consistent. There is considerable individual variation in the problems experienced by patients with CHD and depression. Social problems are central, but PNs and GPs have difficulty in knowing how to address social problems. The person delivering the intervention should understand both CHD and depression. PNs currently manage patients with CHD, but some may need support to manage depression. Since their workload is high, PNs would need convincing that the intervention is feasible and effective. Our empirical work therefore suggested to us that we should develop and test a nurse-led personalised care intervention. We reviewed existing evidence to inform this. This is detailed in Table 1.

**Table 1:** Iterative evidence review: literature used to guide development of an empirically based intervention to improve mood and cardiac outcomes in patients with CHD

Intervention should*	**Findings**	**Conclusions in relation to new intervention**
improve depression, quality of life and cardiac outcomes in patients with CHD	NICE guideline (2009) [16] for depression with a chronic physical health problem recommends a stepped care model involving psychological therapy and/or pharmacotherapy. Two Cochrane reviews [17,18] identify a range of psychological and pharmacological interventions; there is insufficient evidence to determine which elements are beneficial. Psychological interventions and SSRIs were found to improve depression but there was no effect on cardiac outcomes. No specific psychological approach was identified, but larger effect sizes were found for multimodal and collaborative care interventions. A subsequent well conducted trial in the USA[2] found that collaborative care improved depression and disease control in patients with CHD and/or diabetes.	Collaborative care is intensive; our empirical work suggested only a minimal intervention would be feasible. A key ingredient of collaborative care is 'case management' (CM) [19]: a health worker follows up patients, assesses adherence, monitors progress, takes action when treatment is unsuccessful and delivers psychological support [20]. CM is a central aspect of the UK long-term conditions strategy [21] so it is familiar to PNs. CM allows personalised care, but the processes by which change is expected to occur should be specified.
help patients and clinicians to manage an individualised range of problems, including social problems	The CHD and depression association is likely to be explained largely by behaviour [22,23]. Behaviour change interventions for risk behaviours for CHD and depression have been delivered in primary care and shown benefit for smoking [24] and alcohol intake [25]. Not known if they are helpful for patients with CHD and depression. Current UK policy [26] promotes liaison between professionals and utilisation of existing resources to tackle depression and adverse health behaviours.	Training in specific behaviour change techniques and identification of existing local resources, such as social clubs, advice agencies and therapy services, would provide PNs with a ‘toolkit’ of resources which they could tailor according to patient need and preference. Specification of interventions used will inform implementation and evaluation of the intervention.
be nurse-led and feasible for primary care	Case managers are often nurses, but studies lack details concerning implementation and process [27]. Evidence-based behaviour change interventions, such as goal setting and action planning, have been identified for use in primary care [28].	Pilot work should be undertaken to understand which aspects of case management are effective in CHD patients with depression and which outcomes should be targeted.

* We searched for high quality systematic reviews using the Cochrane Library, DARE and NICE guidelines and subsequent evidence using Medline, Embase and Psychinfo. Searches were performed iteratively to build upon initial findings.

Although psychological interventions have been found to improve depression in people with CHD, there is no consensus concerning the most effective approach. However, multi-modal and collaborative care/case management interventions appear to produce the biggest effect sizes [17]. Uncertainty concerning an optimal approach may be due to uncertainty around the mechanisms linking depression and CHD. Physiological mechanisms such as altered inflammatory responses and changes in platelet aggregation have been suggested recently [29], but evidence to date is limited. The influence of health behaviours, such as sedentary lifestyle, unhealthy diet, smoking and poor adherence to exercise or medication regimens however is well established [22,23]. Our empirical work also emphasized the link between depression in patients with CHD and social problems such as loneliness.

Within primary care in the United Kingdom, an established component of chronic disease management is the provision of self management support; this means enabling patients to take better care of themselves, for instance by providing information and helping them to change unhealthy behaviours. Accepted health psychology models agree on two factors important for behaviour change: belief in the importance of an outcome and belief in capacity to succeed (self efficacy) [30]. This suggests that instead of focusing on generic CHD or depression risk factors, the new intervention should enable patients to specify their own goals, for instance stopping smoking or increasing social contact, so that work is directed towards outcomes important to patients. Informed by our evidence review a ‘toolkit’ of behaviour change skills and existing local resources could be developed to facilitate nurses, acting as case managers, to help the patient to increase their self efficacy and achieve their desired outcomes.

We therefore developed a nurse-led personalised care intervention which uses the principles of case management and comprises:

1.) Personalised Care planning: after a standardised biopsychosocial assessment, patients are helped to identify the problems which contribute to depression and CHD and which they choose to work on. Nurses trained in behaviour change interventions supervise the patients' self management of their problems. They also help patients to inform themselves about depression and CHD and contact other relevant agencies such as therapy and social care providers and charities. The specifics of each patient's proposed management is recorded in a care plan which is reviewed regularly.

**Figure 2:**
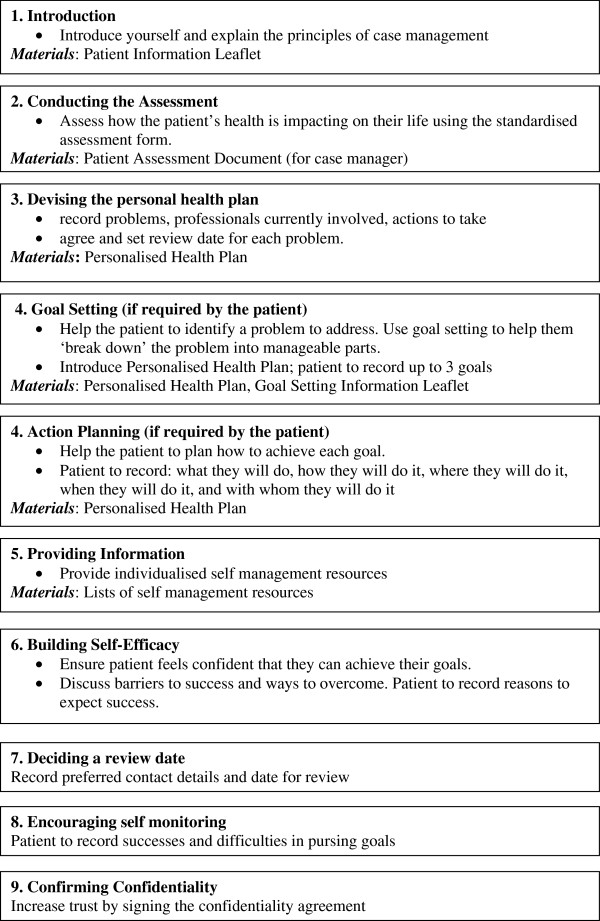
**Personalised care planning.** UPBEAT-UK intervention assessment stage and initial care planning.

**Figure 3:**
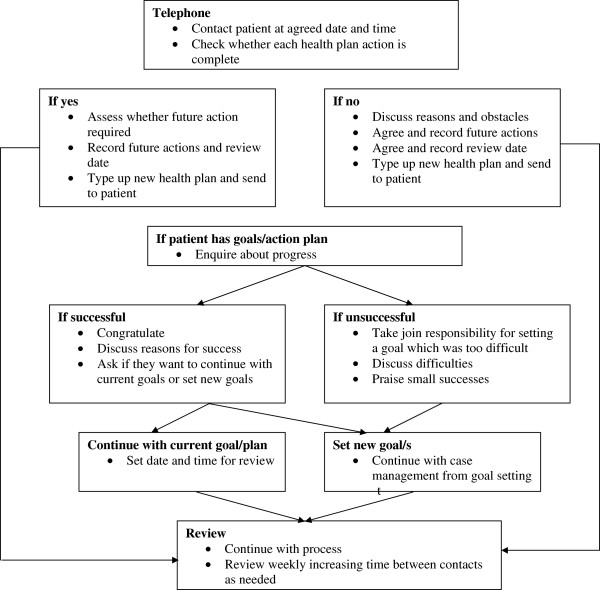
**Follow up care.** UPBEAT-UK intervention follow up care stage.

2.) Follow up care: telephone review is used to conserve time. This is initially weekly and then the frequency of contact is agreed between the patient and nurse case manager. The process is detailed in Figures 2 &3.

### Phase 3: *Modelling of the intervention*

Sixteen patients agreed to take part in the focus group. Thirteen actually participated; 5 out of 6 came for the first group and 8 out of 10 came for the second group. Reasons for not attending were not elicited. Those who participated (53% male) were aged between 48 and 86 years (mean 71). All but two, who were African Caribbean, described themselves as white British.

Aspects of the proposed intervention discussed were (quotes identified by unique patient identification number):

#### Method of delivery

Concern was expressed about the lack of face to face contact with the case manager. Participants agreed that they lack social contact and appeared to want the case manager to provide this.

“You are not having a one to one relationship, you are speaking on the telephone and it seems to me that it is missing the fundamental thing of contact” (murmurs of agreement). (P1049)

However, they agreed that meeting the case manager at least once was important so that they could ‘put a face to the voice’ during telephone follow ups.

#### Duration of intervention

Participants agreed that their problems were likely to be long term.

“I want to be able to see that person for more than three months, they are not going to do nothing to me in three months, I tell you. It probably, it might put me off attempting it if I only know that I have got three months.” (P1060)

They felt that they had high and low periods and wanted support to be available when they felt they needed it.

“It is like I feel good in the past and the slightest things knocks me down. That is why you want someone there to pick you back up again because I can’t pick meself back up.” (P1060)

“A sort of safety net, somewhere that you know that you are not going to fall through” (P1049)

They were also concerned about developing a relationship with someone then being ‘dropped’ once the intervention was over.

“And then you are left with ‘what was that, what was that all about’?” (P1215)

#### Components of the intervention

There were no problems with the assessment form; except that a heading ‘dignity and autonomy’ was not understood. Participants disliked the idea of focusing on only three goals. They felt that they would have provided a lot of information, but that not all of it would be dealt with. Although the participants said they understood the intervention as it was presented on the information sheet, they were more focused on their feelings of loneliness or isolation and most of all wanted someone to talk to.

“It seems to me that it is loneliness is the biggest problem so therefore the contact with a group of people and with a professional would be the answer.” (P1215)

“I feel people need someone to talk to you know, I haven’t got anyone to talk to you know”(P1091)

Participants did not appear to comprehend the intervention aim of enhancing self management.

#### Case managers

Participants of both groups were satisfied that PNs were the correct people to act as case managers because of their understanding of heart disease and its associated problems.

“It would be like counselling by someone who knew the kinds of problems that people would have with a medical condition…and you would talk to somebody like that.” (P1804)

Adaptation of the intervention: The focus group data indicated that patients were very focused on their need for someone to confide in and felt that a case manager would help with this. However, the concept of self management, whereby the case manager would help them to solve their own problems did not seem to be well understood. This will be tested in an exploratory trial. Our plan that nurses should act as case managers, despite the ambivalence identified in our qualitative study of GPs’ and PNs’ views, was supported by our focus group participants who felt that case managers should be able to address physical as well as mental health concerns. Nurses should also be able to provide the continuity of care that participants emphasised as important.

A concern was that some participants’ disliked the planned use of goal setting. Rather, participants were focused on wanting case managers to be a source of social contact; based on our work with PNs this is unlikely to be feasible. Instead, goal setting and action planning could be used to help patients obtain social contact. Furthermore, our literature searches identified good evidence for the feasibility of goal setting in primary care [31]. This highlights the importance, when designing interventions, of utilising multiple sources of evidence. The acceptability of goal setting will be explored further in our exploratory trial and process evaluation.

A minor point that arose from the focus groups was that some of the wording used on the assessment form was not clear. We had developed our care plan format from the framework proposed by the Department of Health as the standard assessment for adults [32,33] this grouped health-related domains such as ‘activities of daily living and mobility’ under headings such as ‘improved personal dignity and autonomy’. These higher level headings appeared to confuse patients, so they were removed and not used in the exploratory trial.

## Discussion

Informed by the MRC framework for the design of complex interventions [6], we conducted empirical studies and iterative literature searching to identify evidence and theory to develop and model a new nurse-led intervention to improve mood and cardiac outcomes in depressed CHD patients.

This approach led us to change our initial plans; based on existing guidelines, we proposed originally to conduct a definitive RCT of a nurse-led stepped care intervention. However, the body of work described here demonstrated clearly a need for an intervention that could be tailored to the differing needs of individual patients. The involvement of PNs in the intervention development was especially useful. That they said they would need support to deliver such an intervention highlighted the need for an exploratory trial to demonstrate to them the feasibility of the intervention.

A potential weakness of this work is that the participants of the patient interview and focus group studies were all drawn from the UPBEAT-UK cohort [3]. Findings from the cohort study have not been published and it is not yet known how representative the participants are of the total population of primary care CHD patients. However, that all the studies reported here were funded by a single programme grant facilitated access to patients and allowed subsequent work to build upon earlier findings in a timely fashion.

Ours is only one possible approach to developing a complex intervention using the MRC framework [6]. A more theory-driven approach would have been to have identified a potentially useful theory at the outset and drafted an intervention as others have done [34] via multidisciplinary discussion. This would have allowed us to test specific theory-related hypotheses. However, we feel that a strength of our approach was that the intervention development was driven by the patients who will receive it and by the clinicians who will deliver it, with theory and evidence used to support choices concerning its content. Ultimately, the success of our approach will be tested in a full RCT of the intervention.

## Conclusions

Our findings, from multiple evidence sources, indicate that a new intervention for CHD patients who are depressed should be flexible enough to address the unique needs important to each patient; it should also utilise existing resources and fit easily into current primary care practice. Application of the MRC framework for complex interventions has allowed us to develop an intervention meeting these criteria which is informed by empirical evidence and established theory. The feasibility and acceptability of this intervention as delivered by research nurses is now being tested in an exploratory trial [35].

## Competing interests

The authors declare no competing interests.

## Authors’ contributions

EB conducted the interviews and the analysis for the staff qualitative study, carried out literature reviews and wrote the first draft of the manuscript. MH also conducted literature searches. RS and ZF conducted the focus group study and its analysis. PW, and AT conceived the studies, assisted in the analysis and interpretation of data and revised the article. JM and DR conceived the qualitative studies, conducted the analysis and revised the article. DR provided service user research expertise. All authors were involved in analyses and iterative literature reviews. All authors read and approved the final manuscript.

## Authors’ information

EB is a practitioner health psychologist, registered general nurse and senior lecturer.

MH is a mental health nurse and senior lecturer.

RS is a social scientist and research worker.

ZF is a social scientist and research worker.

PW is a research fellow and consultant psychiatrist.

JM is a senior lecturer in social research specialising in qualitative studies in mental health.

DM is a Reader in User-Led Research and Head of Section and Co-director Service User Research Enterprise (SURE)

AT is a GP, professor of primary care mental health and Academic Director of the Mood, Anxiety and Personality Clinical Academic Group at Kings’ Health Partners,

King’s College London.

## Pre-publication history

The pre-publication history for this paper can be accessed here:

http://www.biomedcentral.com/1471-2296/13/119/prepub

## References

[B1] Davidson KW Kupfer DJ Bigger JT Califf RM Carney RM Coyne JC Czajkowski SM Frank E Frasure-Smith N Freedland KE Froelicher ES Glassman AH Katon WJ Kaufmann PG Kessler RC Kraemer HC Krishnan KR Lespérance F Rieckmann N Sheps DS Suls JM National Heart, Lung, and Blood Institute Working Group. Assessment and treatment of depression in patients with cardiovascular disease: National Heart, Lung, and Blood Institute working group report". Psychosomatic Medicine 2006 68 5 645 650 10.1097/01.psy.0000233233.48738.22 17012516

[B2] Katon WJ Lin EHB Von Korff M Ciechanowski P Ludman EJ Young B Peterson D Rutter CM McGregor M McCulloch D Collaborative care for patients with depression and chronic illnesses N Engl J Med 2010 363 27 2611 2620 10.1056/NEJMoa1003955 21190455 PMC3312811

[B3] Tylee A Ashworth M Barley E Brown J Chambers J Farmer A Fortune Z Haddad M Lawton R Leese M Mann M Mehay A McCrone P Murray J Pariante C Rose D Rowlands G Smith A Walters P Up-Beat UK: A programme of research in to the relationship between coronary heart disease and depression in primary care patients BMC Fam Pract 2011 12 38 10.1186/1471-2296-12-38 21605435 PMC3120657

[B4] Campbell M Fitzpatrick R Haines A Kinmonth AL Sandercock P Spiegelhalter D Tyre P Framework for design and evaluation of complex interventions to improve health British Medical Journal 2000 321 7262 694 696 10.1136/bmj.321.7262.694 10987780 PMC1118564

[B5] Medical Research Council A Framework for Development and Evaluation of RCTs for Complex Interventions to Improve Health 2000 London, England: Medical Research Council

[B6] Medical Research Council Developing and evaluating complex interventions: new guidance 2008 London, England: Medical Research Council

[B7] Craig P Dieppe P Macintyre S Mitchie S Nazareth I Petticrew M Developing and evaluating complex interventions: the new Medical Research Council guidance BMJ 2008 337 979 983 10.1136/bmj.a979 PMC2769032 18824488

[B8] Barley EA Murray J Walters P Tylee A Managing depression in primary care: A meta-synthesis of qualitative and quantitative research from the UK to identify barriers and facilitators BMC Fam Pract 2011 12 47 10.1186/1471-2296-12-47 21658214 PMC3135545

[B9] Barley EA Walters P Tylee A Murray J General practitioners’ and practice nurses’ views and experience of managing depression in coronary heart disease: a qualitative interview study BMC Fam Pract 2012 13 1 10.1186/1471-2296-13-1 22221509 PMC3276431

[B10] Noblit GW Hare RD Meta-ethnography: synthesizing qualitative studies 1988 London: Sage Publications

[B11] Glaser BG Theoretical sensitivity: Advances in the methodology of grounded theory 1978 Mill Valley CA: Sociology Press

[B12] Braun V Clarke V Using thematic analysis in psychology Qual Res Psychol 2006 3 77 101 10.1191/1478088706qp063oa

[B13] Kroenke K Spitzer RL Williams JB The Patient Health Questionnaire-2: validity of a two-item depression screener Medical Care 2003 2003 41 1284 92 14583691 10.1097/01.MLR.0000093487.78664.3C

[B14] Rose GA The diagnosis of ischaemic heart pain and intermittent claudication in field surveys Bulletin of the World Health Organization 1962 27 645 58 13974778 PMC2555832

[B15] 2NVivo Qualitative data analysis software 2012 Version 10 QSR International Pty Ltd.

[B16] NICE (National Institute for Health and Clinical Excellence) Depression with a chronic physical health problem: the treatment and management of depression in adults with chronic physical health problems. (Partial update of CG23) 2009 http://www.nice.org.uk/CG91

[B17] Baumeister H Hutter N Bengel J Psychological and pharmacological interventions for depression in patients with coronary artery disease Cochrane Database of Systematic Reviews 2011 CD008012 10.1002/14651858.CD008012.pub3 PMC7389312 21901717

[B18] Rees K Bennett P West R Davey SG Ebrahim S Psychological interventions for coronary heart disease Cochrane Database of Systematic Reviews 2004 2 CD002902 10.1002/14651858.CD002902.pub2 PMC4170898 15106183

[B19] Gilbody S Bower P Fletcher J Ricahrds D Sutton AJ Collaborative care for depression: a cumulative meta-analysis and review of longer-term outcomes Arch Intern Med 2006 166 2314 212 10.1001/archinte.166.21.2314 17130383

[B20] Von Korff M Goldberg DP Improving outcomes in depression British Medical Journal 2001 323 948 949 10.1136/bmj.323.7319.948 11679372 PMC1121496

[B21] Department of Health Improving Chronic Disease Management 2004 London: Department of Health

[B22] Hamer M Molloy GJ Stamatakis E Psychological Distress as a Risk Factor for Cardiovascular Events: Pathophysiological and Behavioral Mechanisms J Am Coll Cardiol 2008 52 25 2156 2162 10.1016/j.jacc.2008.08.057 19095133

[B23] Whooley MA de Jonge P Vittinghoff E Otte C Moos R Carney RM Ali S Dowray S Na B Feldman MD Schiller NB Browner WS Depressive Symptoms, Health Behaviors, and Risk of Cardiovascular Events in Patients With Coronary Heart Disease JAMA 2008 300 20 2379 2388 10.1001/jama.2008.711 19033588 PMC2677371

[B24] Stead LF Bergson G Lancaster T Physician advice for smoking cessation Cochrane Database of Systematic Reviews(2) 2008 Art. No.: CD000165 10.1002/14651858.CD000165.pub3 18425860

[B25] Kaner EF Beyer F Dickinson HO Pienaar E Campbell F Schlesinger C Heather N Saunders J Burnand B Effectiveness of brief alcohol interventions in primary care populations (Review) Cochrane Database of Systematic Reviews(2) 2007 Art. No.: CD004148 10.1002/14651858 17443541

[B26] Department of Health Healthy lives, Healthy people 2011 London: Department of Health http://www.dh.gov.uk/prod_consum_dh/groups/dh_digitalassets/documents/digitalasset/dh_129334.pdf

[B27] Reilly S Hughes J Challis D Case management for long-term conditions: implementation and processes Ageing and Society 2010 30 125 155 10.1017/S0144686X09990183

[B28] Michie S Rumsey N Fussell A Hardeman W Johnston M Newman S Yardley L Improving health: changing behaviour. NHS health trainer handbook. Manual 2008 Department of Health Publications (Best Practice Guidance: Gateway Ref 9721)

[B29] Pariante CM Lightman SL The HPA axis in major depression: classical theories and new developments Trends Neurosci 2008 31 464 468 10.1016/j.tins.2008.06.006 18675469

[B30] Spanou C Simpson SA Hood K Edwards A Cohen D Rollnick S Carter B McCambridge J Moore L Randell E Pickles T Smith C Lane C Wood F Thornton H Butler CC Preventing disease through opportunistic, rapid engagement by primary care teams using behaviour change counselling (PRE-EMPT): protocol for a general practice-based cluster randomised trial BMC Fam Pract 2010 11 69 10.1186/1471-2296-11-69 20858273 PMC2955601

[B31] Bodenheimer T Handley MA Goal-setting for behavior change in primary care: An exploration and status report Patient Education and Counseling 2009 76 174 180 10.1016/j.pec.2009.06.001 19560895

[B32] Department of Health Independence, Well-being and Choice: Our Vision for the Future of Social Care for Adults in England 2005 London: Department of Health

[B33] Department of Health Common Assessment Framework for Adults: a consultation on proposals to improve information sharing around multi-disciplinary assessment and care planning 2009 London: Department of Health

[B34] Smith SM Murchie P Devereux G Jahnston M Lee AJ Macleod U Nicolson MC Powell R Ritchie LD Wyke S Campbell NC Developing a complex intervention to reduce time to presentation with symptoms of lung cancer BJGP 2012 62 602 605 15 10.3399/bjgp12X654579 22947581 PMC3426599

[B35] Tylee A Haddad M Barley E Ashworth M Brown J Chambers J Farmer A Fortune Z Lawton R Leese M Mann A McCrone P Murray J Pariante C Phillips R Rose E Rowlands G Sabes-Figuera R Smith A Walters P A pilot randomised controlled trial of personalised care for depressed patients with symptomatic coronary heart disease in South London general practices: the UPBEAT-UK RCT protocol and recruitment BMC Psychiatry 2012 12 58 10.1186/1471-244X-12-58 22672407 PMC3437191

